# Supplementary material for: What is the contribution of physician associates in hospital care in England? A mixed methods, multiple case study
Source: BMJ Open. 2019 Jan 30;9(1):e027012. doi: 10.1136/bmjopen-2018-027012 (PMC6359738; doi:10.1136/bmjopen-2018-027012)
Supplement: Supplementary data [file bmjopen-2018-027012supp001.pdf]

Supplementary file 1

Consensus standards for the reporting of organisational case studies

Reporting item	Page number on which item was reported	Page number of justification for not reporting
**Describing the design**		
1. Define the research as a case study	6	
2. State the broad aims of the study	5	
3. State the research question(s)/hypotheses	5 & 6	
4. Identify the specific case(s) and justify the selection		
**Describing the data collection**		
5. Describe how data were collected	6 & &	
6. Describe the sources of evidence used	6 & 7	
7. Describe any ethical considerations and obtainment of relevant approvals, access and permissions	7	
**Describing the data analysis**		
8. Describe the analysis methods	7	
**Interpreting the results**		
9. Describe any inherent shortcomings in the design and analysis and how these might have influenced the findings	17	
10. Consider the appropriateness of methods used for the question and subject matter and why it was that qualitative methods were appropriate	16 & 17	
11. Discuss the data analysis	16 & 17	
12. Ensure that the assertions are sound, neither over- nor under-interpreting the data	17	
13. State any caveats about the study	17	

From: Chapter 5, Translating high-consensus Delphi items into reporting standards for organisational case studies Developing a methodological framework for organisational case studies: a rapid review and consensus development process. Health Services and Delivery Research, No. 4.1. Rodgers M, Thomas S, Harden M, et al. Southampton (UK): NIHR Journals Library; 2016 Jan
