# Supplementary material for: What is the contribution of physician associates in hospital care in England? A mixed methods, multiple case study
Source: BMJ Open. 2019 Jan 30;9(1):e027012. doi: 10.1136/bmjopen-2018-027012 (PMC6359738; doi:10.1136/bmjopen-2018-027012)
Supplement: Supplementary data [file bmjopen-2018-027012supp002.pdf]

Supplementary material 2

Topics guides for semi-structured interviews

Topic guide for senior managers and clinicians

Topic areas

- Confirm the person's job role
- Ask them to describe their involvement with physician associate employment in the hospital to date
- Ask questions on the factors supporting the adoption of the employment of physician associates
- Ask questions on the factors inhibiting the employment of physician associates
- Questions on their views of physician associates' impact on (ask for examples):
  - Organisation of services
  - Patient experience and outcomes
  - Other staff
  - Costs
- Anything else they would like to say?

Interviewer to probe on all answers to ensure the meaning is clear (e.g. that is an interesting point , can you explain a bit more about it) and check for understanding (e.g. so can I check I have understood you correctly)…….

Interviewer to check for any routine management reports or data or evaluations that the hospital team would be willing to share with the researchers.

Thank them and ask if they would like to receive updates on the study and a final summary of the findings. If so could they please give contact details which will be kept separate from the interview data?

Topic guide for physician associate interviews

<u>Topic areas</u>

- Ask them to describe how long they have been a physician associate, how many posts, type and length as a physician associate
- Ask them to describe the work they undertake, with what type of medical/surgical team
- Ask about their supervising doctor and arrangements when they are not there
- Ask questions on their views of the factors supporting the adoption of the employment of physician associate in their experience
- Ask questions on their views of the factors inhibiting the employment of physician associate in their experience
- Ask how they have been received in the hospital as a new type of health professional?
- Ask how they explain to patients, family and staff – who they are and what a physician associate is

- Questions on their views of their, or other physician associates, impact on (ask for examples):
    - Organisation of services
    - Patient experience and outcomes
    - Other staff
    - Costs

- Anything else they would like to say?

Interviewer to probe on all answers to ensure the meaning is clear (e.g that is an interesting point , can you explain a bit more about it) and check for understanding (e.g. so can I check I have understood you correctly)…….

Interviewer to check for any routine management reports or data or evaluations that the hospital team would be willing to share with the researchers .

Thank them and ask if they would like to receive updates on the study and a final summary of the findings. If so could they please give contact details which will be kept separate from the interview data?

Topic guide for all other types of professionals/managers interviews

Topic areas

- Confirm the person's job role
- Ask them to describe their involvement with physician associate employment in the hospital to date
- Ask questions on their views of any factors supporting the adoption of the employment of physician associates in their experience
- Ask questions on their views of any factors inhibiting the employment of physician associates in their experience
- Ask their views as to how the PAs have been received in that service/team, and probe for any explanations
- Questions on their views of physician associates' impact on (ask for examples):
    - Organisation of services
    - Boundaries between the job roles of different types of professionals e.g. with nurses
    - Patient experience and outcomes
    - Other staff
    - Costs

- Anything else they would like to say?

Interviewer to probe on all answers to ensure the meaning is clear (e.g. that is an interesting point , can you explain a bit more about it) and check for understanding (e.g. so can I check I have understood you correctly)…….

Interviewer to check for any routine management reports or data or evaluations that the hospital team would be willing to share with the researchers.

Thank them and ask if they would like to receive updates on the study and a final summary of the findings. If so could they please give contact details which will be kept separate from the interview data?

Topic guide for patient interviews

Topic areas

- Confirm the person is/has been a patient
- Ask them to outline the type of care they have been in receipt of without giving personal medical details e.g. in patient for x days
- Confirm the patient has met the physician associate
- Explore what sort of involvement the physician associate has had with them
- Ask them how they understand the role of the physician associate in the medical/surgical team
- Ask them how they found receiving care from a physician associate
- If they were to need similar medical or surgical care, would they be content to receive similar care from a physician associate in the future as they had this time (and can they explain why) or would they prefer someone different? And if yes, can they explain why?
- Anything else they would like to say?

Interviewer to probe on all answers to ensure the meaning is clear (e.g. that is an interesting point , can you explain a bit more about it) and check for understanding (e.g. so can I check I have understood you correctly).

Thank them and ask if they would like to receive updates on the study and a final summary of the findings. If so could they please give contact details which will be kept separate from the interview data?
